# Mucocele of the appendix – a diagnostic dilemma: a case report

**DOI:** 10.1186/1752-1947-1-183

**Published:** 2007-12-19

**Authors:** Ciprian Bartlett, Madhavi Manoharan, Anne Jackson

**Affiliations:** 1 Department of Obstetrics and Gynaecology, Homerton University NHS Foundation Trust, London, UK; 2 Consultant Obstetrician and Gynaecologist, Barnet and Chase Farm Hospital NHS Trust, The Ridgeway, Enfield, EN2 8JL, UK; 3 Department of Women and Children, Homerton University Hospital, Homerton Row, London, E9 6SR, UK

## Abstract

**Introduction:**

Mucocele of the appendix secondary to mucinous cystadenoma is a rare clinical finding. Clinical presentation is varied with more than half being asymptomatic.

**Case presentation:**

We report such a case presenting to the surgeons where initial clinical findings and investigations suggested an ovarian cyst. The patient was subsequently referred to the Gynaecologists for further management. In spite of extensive preoperative investigations, the diagnosis was only made at the time of surgery.

**Conclusion:**

In women presenting with a right iliac fossa mass and clinical features not indicative of gynaecological pathology, an appendiceal origin should be considered in the differential diagnosis.

## Introduction

Mucocele of the appendix secondary to mucinous cystadenoma is a rare clinical finding and we report such a case presenting in a district general hospital. They can present as a pelvic mass and thus pose a diagnostic challenge.

Currently, the assessment of pelvic masses relies heavily on USS as the primary diagnostic tool. This however may not always identify the origin of such a mass. In such cases, clinical findings and other investigative modalities are warranted to aid the diagnostic process. In spite of extensive preoperative investigations, the diagnosis may still remain elusive and may only be made at the time of surgery.

## Case presentation

An eighty year old woman was referred to the General Surgeons with right sided abdominal pain and weight loss over several months. There was no associated urinary or bowel symptoms. On examination, there was clinical evidence of weight loss with a suggestion of a fixed right sided pelvic mass per rectum. The CA 125 was within normal limits. An ultrasound scan showed a right sided mixed echogenic pelvic mass with an echogenic rim, possibly ovarian in origin, measuring 61 × 43 × 51 mm. A CT of the abdomen and pelvis suggested a calcified adnexal cyst 7 × 6 × 5 cm with no evidence of lymphadenopathy and she was referred to the Gynaecologist. When reviewed by the Gynaecologist, no mass was palpable per abdomen or per vaginum. She had an exploratory laparotomy where the only pathology identified was a distended appendix and a routine appendicectomy was performed. Histology showed mucocele of the vermiform appendix secondary to mucinous cystadenoma.

## Discussion

Mucocele of the appendix is a descriptive term for an appendix distended by mucus, secondary to mucinous cystadenoma (63%), mucosal hyperplasia (25%), mucinous cystadenocarcinoma (11%) and retention cyst [1].

Mucocele can also occur due to occlusion of the lumen by endometriosis or carcinoid tumour.

Overall, appendiceal mucoceles make up about 0.2%–0.3% of appendix specimen. Clinical presentation may include right lower quadrant pain, change in bowel habits, per rectal bleeding or a palpable mass [2]. Approximately 23–50% of patients are asymptomatic, with the lesions being discovered incidentally during surgery, radiological evaluations or endoscopic procedures [2-4]. In our case, it is likely that the symptoms of right lower quadrant pain and weight loss were not related to the mucocele since this benign mass was not tender on palpation. In addition, the symptoms did not assist in making the pre-operative diagnosis. The preoperative clinical diagnosis of appendiceal mucoceles can therefore be difficult because of this lack of clinical symptomotology.

The initial detection of the lesion may be facilitated by radiological, sonographic or endoscopic means.

On barium enema, there is usually non filling or partial filling of the appendix with contrast. The lesion may be seen as a sharply outlined sub mucosal or extrinsic mass indenting the caecum and laterally displacing it [3].

CT of the abdomen usually shows a cystic well-encapslated mass sometimes with mural calcification, in the expected location of the appendix. It may be causing extrinsic pressure on the caecal wall without any surrounding inflammatory reaction [3,5-7].

Ultrasound findings can be variable. Purely cystic lesions with anechoic fluid, hypoechoic masses with fine internal echoes as well as complex hyperechoic masses can be seen depending on the contents [8]. The onion skin sign is considered to be specific for mucocele of the appendix [9].

Colonoscopic findings include the 'volcano sign', the appendiceal orifice seen in the centre of a firm mound covered by normal mucosa or a yellowish, lipoma-like submucosal mass [10].

In the above case report, USS and CT were unable to provide a preoperative diagnosis. The clinical suspicion of gastrointestinal pathology due to lack of pelvic findings, more closely correlated to the operative findings.

In our case, the decision for excision of the appendiceal mucocele was made as a result of diagnostic uncertainty and a need to rule out malignancy.

Surgical excision of mucocele of appendix can either be by laparotomy or laparoscopy. Laparoscopic surgery provides the advantages of good exposure and evaluation of entire abdominal cavity, as well as more rapid recovery with avoidance of a large incision and a better cosmetic outcome. However careful handling of the specimen is recommended as spillage of the contents can lead to pseudomyxoma peritonei. This can be achieved by atraumatic handling of the appendix and use of impermeable bag for removal of the specimen. Conversion to laparotomy should be considered if the lesion is traumatically grasped or if the tumour clearly extends beyond the appendix or if there is evidence of malignancy such as peritoneal deposits [11]. Involvement of the caecum or adjacent organs is an indication for right hemi-colectomy and thorough exploration of the gastrointestinal tract and ovaries [12].

## Conclusion

Mucocele of the appendix can mimic an adnexal mass and prove to be a diagnostic challenge. In a woman presenting with right iliac fossa mass and with clinical features not indicative of gynaecological pathology, an appendiceal origin should be considered in the differential diagnosis.

## Abbreviations

CA 125 – Cancer Antigen 125

CT – Computerised Tomography

CEA – Carcino-Embryonic Antigen

USS – Ultrasound Scan

## Competing interests

The author(s) declare that they have no competing interests.

## Authors' contributions

CEB – Literature review, conceived and drafted the manuscript.

MM – Helped in collecting the records and preparing the manuscript.

AEJ – Department chair who provided general support.

All the authors revised and approved the manuscript.

## Consent

Written informed consent was obtained from the patient for publication of this case report and any accompanying images. A copy of the written consent is available for review by the Editor-in-Chief of this journal.
